# On the understanding and development of modern physical neurorehabilitation methods: robotics and non-invasive brain stimulation

**DOI:** 10.1186/1743-0003-6-3

**Published:** 2009-01-30

**Authors:** Dylan J Edwards

**Affiliations:** 1 Burke Medical Research Institute, White Plains, NY, USA; 2 Department of Neurology & Neuroscience, Weill Medical College, Cornell University, White Plains, NY, USA; 3 Berenson-Allen Center for Non-Invasive Brain Stimulation, Harvard Medical School, Boston, MA, USA; 4 Centre for Neuromuscular and Neurological Disorders, University of Western Australia, Australia

## Abstract

The incidence of physical disability in the community resulting from neurological dysfunction is predicted to increase in the coming years. The impetus for immediate and critical evaluation of physical neurorehabilitation strategies stems from the largely incomplete recovery following neurological damage, questionable efficacy of individual rehabilitation techniques, and the progressive acceptance of evidence-based medicine. The emergent technologies of non-invasive brain stimulation (NBS) and rehabilitation robotics enable a better understanding of the recovery process, as well as the mechanisms and effectiveness of intervention. With a more precise grasp of the relationship between dysfunctional and treatment-related plasticity, we can anticipate a move toward highly controlled and individualised prescription of rehabilitation. Both robotics and NBS can also be used to enhance motor control and learning in patients with neurological dysfunction. The merit of these contemporary methods as investigative and rehabilitation tools requires clarification and discussion. In this thematic series, five cohesive and eloquent papers address this issue from leading clinicians and scientists in the fields of robotics, NBS, plasticity and motor learning.

## Introduction

There is a pressing need to improve physical neurorehabilitation strategies. This might be accomplished by learning from observations of accurately defined physiological and behavioural aspects of motor recovery. Technologies such as non-invasive brain stimulation (NBS) and robotics can provide sensitive and reliable measures to achieve this, and might additionally be incorporated as therapeutic tools to augment rehabilitation strategies. While these techniques are still developing, over a decade of scientific work is uncovering their mechanisms and efficacy. To the rehabilitation practitioner, this literature may seem esoteric and far removed from daily practice. This thematic series brings together leaders in the field of robotics, NBS and plasticity, to provide an up-to-date overview for scientists and clinicians interested in physical neurorehabilitation.

## Physical neurorehabilitation in context

The early 21^st ^century may be an important time in the advancement of the medical field, where practice not based on credible scientific evidence is increasingly questioned. As well, this time might be characterized by rapid and global dissemination of research findings, and the emergence of technologies that may translate into significant improvements in preventative medicine and acute care. Yet, according to the World Health Report 2008, we can anticipate an unprecedented impact of diseases resulting from our progressively ageing population [1]. A prominent contributor to the future burden of disease will be cerebrovascular disease, or stroke, as well as neurodegenerative disorders. Over the next several decades, the cost of stroke is expected to exceed a trillion dollars in the US alone [2]. So what can be done now to mitigate this? Since a large part of the cost of stroke is the management of ongoing disability, targeted and effective rehabilitation could potentially reduce this burden. But does rehabilitation work? How does it work? In stroke rehabilitation, a number of restorative therapies currently exist and others are in various stages of evolution (for review see [3]). Physical therapies are a major component of post-stroke rehabilitation because reduced motor function influences the ability of patients to perform activities of daily living, and has orthopaedic, cardiorespiratory and cerebrovascular implications. However, there are reservations in the medical community concerning the benefit of various types of physical therapies.

## Evolution of physical rehabilitation

Historically, the management of stroke survivors involved bed-rest and convalescence, reminiscent of the familiar quote attributed to Voltaire (1694–1778); "the role of the physician is to entertain his patient while nature takes it course". The concept of physical therapies developed through the early to mid 1900s, resulting largely from efforts to help world-war and polio survivors. The branch of medicine devoted to active rehabilitation was formed, and a shift from 'do nothing' to 'do something' occurred. This was based on the understanding of neurological dysfunction at the time, and the observation that patients appeared to tolerate and benefit from physical interventions. Observations that patients had better outcomes if exposed to 'enriched environments' versus bed-rest were increasingly reported. However the critical aspects of the enriched experience remained an enigma. Distinct approaches to rehabilitation practice emerged in parallel to increasing understanding of the human nervous system and the response to physical interventions. These approaches had different characteristics which might be loosely summarized as focusing on the following outcomes: impairment reduction, suppression of muscle tone and restoration of normal movement, or repetitive training to improve function. As hospitals and care facilities world-wide increasingly incorporated physical therapies as routine management for neurological patients, there was a move from 'do something', to 'do something *specific*'. But there was debate amongst rehabilitation professionals about the most effective type and amount of therapy. Presently, the superiority of the various techniques still remains questionable when held to scientific scrutiny.

## Is physical rehabilitation effective?

A recent review of randomized controlled trials in physical therapy treatment alternatives (in over 1000 stroke patients), indicates that physical therapies provide improvement in function when compared to no treatment or control [4]. Yet importantly, superiority of one type of therapy over another could not be distinguished, and therefore that the specific choice of therapy was difficult to justify over another type of treatment. This concept has been similarly described by others [5]. So, building on our evolution of physical therapies, have we now progressed from 'do nothing', to 'do something', to 'do something specific', and finally to 'do *anything*'? This is of course impudent since informed and experienced practitioners intelligently prescribe safe and appropriate therapies leading to clinically meaningful improvements. But what makes therapies effective? Was Voltaire right? Do we serve to 'entertain' or perhaps more appropriately 'encourage' or 'motivate'? There is clearly something about the psychosocial nature of regular interaction with health professionals that is beneficial, but also that active, motivated engagement in activity leads to higher levels of function. If the benefit is to extend beyond orthopaedic, and impact recovery of motor control, we know that therapies cannot be passive and patients must be engaged. Perhaps part of the 'magic' in the hands of the individual therapist might be the ability to engage the patient and direct appropriate attention to task.

It is apparent then, that focused guided rehabilitation efforts, performed repetitively, result in improved motor function together with the decreased risk of secondary health problems. Yet this is labour intensive. Furthermore, patients often find the training uninteresting, tiring and lacking meaning for them. Robotic or Virtual Reality technology may overcome these barriers by making physical rehabilitation interesting, challenging and even addictive, all perhaps necessary ingredients for relearning motor skills after neurological damage. There is clearly a need for optimising rehabilitation strategies to provide cost efficient, fun, effective methods that maximize long-term health and function. The scientific challenge is to build on existing technologies and strategies to achieve this important outcome.

## Understanding rehabilitation interventions

In order to progress from current rehabilitation strategies, we need to have a solid and precise understanding of specific therapies. There is a need for research into the effectiveness of *clearly-described *physical rehabilitation methods, at a specific time for specific patients. Rehabilitation robotics may represent the most sophisticated method available today for reliably and precisely driving therapeutic engagement and measuring precise outcomes. Robotic technology can be used to quantify and track motor behaviour for individual patients. Kinematic and kinetic data can be obtained during therapy sessions or during a separate evaluation, thus making rehabilitation robots an ideal tool to provide motor control measures – more sensitive and reliable than standard clinical scales. In addition to helping us understand motor control in relation to specific lesion and individual characteristics, robotics can help us understand the effectiveness of motor learning paradigms. Robotics can be used to investigate if patients recovering from neurological lesion might acquire motor skills similar to healthy adults. Huang and Krakauer present a paper titled 'Robotic Neurorehabilitation: A Computational Motor Learning Perspective' which provides a comprehensive and lucid discussion of what is currently known about return of motor function following neurological damage, and what we can take from computational motor learning literature to set up training paradigms using robotic devices that might optimally promote development and retention of motor skills and translate into long-term reduction in disability.

Also supporting a quantitative scientific understanding of mechanisms of post-stroke recovery, Krebs, Volpe, and Hogan present a paper titled 'A Working Model of Stroke Recovery from Rehabilitation Robotics Practitioners'. Their perspective is based on experience with the implementation of rehabiltitation robotics in a large number of stroke patients, and they present data supporting a model of motor recovery post-stroke, resulting from their experiences. They propose that coordination training may be most the beneficial form of intervention and may lead to motor relearning, while resistive training is only beneficial in some cases, and passive training resulted in no motor learning.

## Can we augment recovery and plasticity with brain stimulation?

One of the contemporary methods under investigation as a tool to promote neuroplasticity is non-invasive brain stimulation. The concept of treating neurological disorders with electricity is not new, and despite an interesting history dating back to ancient times, was considered to be first seriously attempted in the mid 1700s; perhaps the start of medical electrotherapy [6]. At this time, it was thought that it may be potentially useful for improving function following stroke. However despite continued attempts over the next 200 years, the evidence for efficacy of this treatment was sketchy and the methods limited incorporation into neurological practice. The development of commercially available non-invasive brain stimulators in the late 1900s again sparked interest, initially in the use of this tool for testing the integrity of the nervous system, and later for neuromodulation. Following the early papers reporting that TMS applied repetitively could lead to a change in cortical excitability – outlasting the stimulation period – the focus of research became to understand parameters of stimulation (e.g. intensity, duration, frequency) that might lead to desired effects (increase or decrease in excitability) in the desired location [7]. Also, which parameters gave the most long-lasting effects, and whether these effects lead to any clinically meaningful changes in function for a variety of neurological disorders. Such investigations are still underway and are typically applied with the subject at rest. However the concept of coupling NBS with deliberate voluntary brain activity has only recently gained interest. Can the interaction of transient excitability changes from NBS support the changes induced by motor practice paradigms to augment motor learning? Bolognini, Pascual-Leone and Fregni provide an overview of this contemporary topic in the paper 'Using non-invasive brain stimulation to augment motor training-induced plasticity'. A discussion of the rationale for combining NBS with purposeful behavioural training is provided, and they suggest that different NBS protocols may be required at specific times post stroke, and also in relation to a given physical training session (e.g. before, during or after motor training). They propose that stimulation during training may be most effective and discuss the importance of the nature of the specific *training *paradigm in relation to a given *stimulation *paradigm. The authors suggest that the combination of NBS with functional therapies has the potential to drive plastic changes in brain-damaged patients, only if guided by a careful consideration of underlying mechanisms. This is important, since combined therapies may not necessarily be complementary.

One premise of NBS as a potential adjuvant for therapy is that it induces plasticity and that plasticity is important and helpful. While evidence exists that NBS in humans can be beneficial, the circumstances under which it works most effectively and the mechanisms are not fully understood. Huerta and Volpe address potential mechanisms in their paper 'Transcranial magnetic stimulation, synaptic plasticity and network oscillations'. The paper provides insight from basic sciences literature about cellular mechanisms of plasticity, including an apposite discussion of processes leading to potentiation and depression of synaptic strength. The similarity of human NBS protocols to long-term-potentiation (LTP) and long-term-depression (LTD) protocols in animal studies is described, as well as implications for interpreting studies of NBS in human motor cortex. Successful therapeutic application of NBS techniques in humans additionally requires understanding the relationship of functional and dysfunctional plasticity across different neurological conditions. This is true even within a given condition, such as stroke, which itself represents a diverse mix of injury type, clinical presentation and recovery prognosis. Thickbroom and Mastaglia outline important considerations for advancing NBS as a potential therapeutic tool in disorders of the brain and spinal cord in a paper titled 'Plasticity in neurological disorders and challenges for noninvasive brain stimulation'. The paper describes how plasticity is implicated in neurological disorders, and discusses current NBS plasticity protocols applied in clinical research.

## Understanding stroke recovery

This thematic series emphasizes the understanding and development of modern neurorehabilitation methods, and outlines some ways to quantify plasticity and recovery of function. However, we are only beginning to understand the complex interactions affecting recovery from acute brain injury (for review see [8]). The multifactorial nature of recovery from neurological lesion will no doubt influence the success of intervention paradigms. One of Time Magazine's 2008 most influential scientists and thinkers, neuroscientist and stroke survivor Dr Jill Bolte Taylor claims that sleep is the number one consideration for recovery after stroke [9,10], and that we now do too much therapy with not enough sleep. Perhaps sleep is important even beyond the rehabilitation hospital and into the chronic phase. We know that retention of transient practice effects requires a consolidation period that involves sleep, and stroke patients deprived of sleep fail to retain implicit motor skill [11]. So while we may need specific therapy at specific times for specific patients, we also need to 'do nothing' at times. The timing and amount of both training and sleep requires further investigation. Other questions arise from current literature, for instance why do comparable level patients of similar age exposed to the same therapy or intervention respond differently? Some patients may be genetically predisposed to adapt better to intervention [12], and this raises the question of whether we should give different therapies based on genetic profile? This also provides promise for potential targets of pharmacologic and genetic manipulation. Future studies of robotics and NBS should systematically address these and other factors affecting recovery in relation to specific interventions.

## Conclusion

The rapid advancement in technology, together with more rigid clinical experimental protocols, will enable a better understanding of physical rehabilitation therapies. Coupled with an increasing knowledge of plasticity in neurological disorders, we can expect that rehabilitation practices and treatments will continue to evolve in parallel. The type and schedule of rehabilitation intervention may ultimately be prescribed based on detailed characteristics of the individual, and the time since lesion or state of the disease. I refer readers to the individual papers in this thematic series of JNER for an insightful and thorough account of the basis for these evolving practices.
